# Exploring University Instructors’ Achievement Goals and Discrete Emotions

**DOI:** 10.3389/fpsyg.2020.01484

**Published:** 2020-08-07

**Authors:** Raven Rinas, Markus Dresel, Julia Hein, Stefan Janke, Oliver Dickhäuser, Martin Daumiller

**Affiliations:** ^1^Department of Psychology, University of Augsburg, Augsburg, Germany; ^2^Department of Educational Psychology, University of Mannheim, Mannheim, Germany

**Keywords:** higher education, university instructors, achievement goals, discrete emotions, goal orientation, motivation

## Abstract

Emerging empirical evidence indicates that discrete emotions are associated with teaching practices and professional experiences of university instructors. However, further investigations are necessary given that university instructors often face high job demands and compromised well-being. Achievement goals, which frame achievement-related thoughts and actions, have been found to describe motivational differences in university instructors and are hypothesized to be associated with their discrete emotions. Moreover, as variation exists in how university instructors respond to job demands regarding their emotional experiences, certain goals may moderate this relationship on the basis of framing different interpretations and reactions to stressors. To investigate these links, 439 instructors (46.7% female) from German and Austrian universities completed a survey assessing their achievement goals, discrete emotions (enjoyment, pride, anger, anxiety, shame, and boredom), and job demands. As hypothesized, multiple regression analyses revealed that achievement goals were differentially and meaningfully associated with discrete emotions. Specifically, learning approach goals were positively related to enjoyment and negatively related to anger and boredom, while learning avoidance goals were positively related to anger. Performance (appearance) approach goals were positively related to pride, and performance (appearance) avoidance goals were positively related to anxiety and shame. Lastly, relational goals were positively related to shame and boredom, and work avoidance goals were negatively related to enjoyment and positively related to shame and boredom. Conclusive moderation effects on the relations between job demands and emotions were not found. Future research avenues aimed at further understanding the supportive role that achievement goals can have for university instructors’ emotional experiences and well-being are discussed.

## Introduction

A wealth of empirical evidence indicates that teachers’ emotions contribute to important outcomes such as their instructional quality and well-being (Frenzel et al., 2009, 2016; Yin et al., 2018; Chen, 2019). Many of these studies have focused on school teachers, while research examining university instructors’ emotions is still in its early developmental stages. The few existing studies have consistently found that university instructors’ emotions matter, having been associated with professional and personal balance (Stupnisky et al., 2019a), perceived teaching success (Stupnisky et al., 2019b), and appraisals of teaching value and self-determination (Thies and Kordts-Freudinger, 2019). These findings are especially relevant as research suggests that university instructors face high job demands and compromised well-being (e.g., Bell et al., 2012; Mudrak et al., 2018).

Consequently, further investigations into the antecedents of university instructors’ emotions are needed, not only to better understand how to support instructor well-being, but also for the students and academic institutions who depend on them. From a motivational perspective, achievement goals, which frame one’s cognitive, affective, and behavioral processes in achievement settings (Nicholls, 1984; Dweck, 1986; Ames, 1992; Elliot and Church, 1997), have been highlighted as antecedents of discrete emotions in students (e.g., Pekrun et al., 2006; Schutz and Pekrun, 2007) and in school teachers (Wang et al., 2016; Janke et al., 2019a). However, to the best of our knowledge, no studies to date have explored these relations in university instructors—a population who, given their unique and important role in society, require specific reserach attention (Daumiller et al., 2020b).

Adding to this, research has shown that university instructors are confronted with high job demands and, in turn, may experience adverse outcomes such as negative work-related emotions (Mudrak et al., 2018). However, not all instructors experience these outcomes to the same extent (see Shin and Jung, 2014). To better understand this variation, identifying variables that alter the strength or direction of the relationship between job demands and emotions marks a promising research direction. Given that the pursuit of certain achievement goals—especially learning approach and work avoidance goals—may suggest different perceptions of job demands, we examined these goals as theoretically plausible moderators. Specifically, learning approach goals may lead university instructors to perceive job demands as learning opportunities, while work avoidance goals may lead them to perceive job demands as stressful experiences to avoid.

Taken together, the primary aim of the present study was to investigate the associations between university instructors’ achievement goals for teaching and their discrete emotions. To provide a thorough overview of these relations, we rely on an achievement goal model that summarizes the most relevant theoretically distinguishable achievement goal classes found for this population (Daumiller et al., 2019b). We additionally examined the role of learning approach and work avoidance goals as moderators in the relationship between job demands and discrete emotions.

### University Instructors’ Emotions

University instructors’ emotional, affective, and well-being experiences are becoming trending topics within educational research (see special issues of Kinman and Johnson, 2019; Mendzheritskaya and Hansen, 2019; Daumiller et al., 2020b). In the present study, we are particularly interested in their discrete emotions, which describe emotions that are separable and distinct from one another, such as enjoyment or anger. This is in contrast to, for example, positive or negative affect, which refer to more general level emotional experiences. Prior research has argued that there is a lack of studies examining university instructors’ discrete emotions (see Stupnisky et al., 2019a) and that not all discrete emotions within the categories of positive and negative affect necessarily share the same associations with different teaching-related variables (see Frenzel et al., 2016; Lee and van Vlack, 2017). Therefore, taking a discrete perspective affords more detailed information concerning university instructors’ emotions. In particular, we examined the emotions of enjoyment, pride, anger, anxiety, shame, and boredom, which have been found to be especially relevant and frequently experienced by university instructors in the teaching domain, as highlighted in the following literature review.

Although empirical evidence on university instructors’ discrete emotions is limited, two central research areas can be distinguished within the literature. The first constitutes gaining knowledge about the relevance and frequency of university instructors’ emotions, while the second entails quantitatively examining how these emotions are related to other relevant variables. Some studies also focus on the emotions of early-career faculty (e.g., Stupnisky et al., 2016, 2019a, 2019b), suggesting that they may experience more stress than their senior counterparts as they are still adjusting to the demanding faculty lifestyle (Stupnisky et al., 2016). In this light, it is important to mention that in the higher education systems in Germany and Austria where the current study took place, university faculty (irrespective of their rank) typically have both teaching and research responsibilities in their employment contract. In these contexts, it is also common for doctoral candidates to be hired as university faculty members. Thus, alongside fulfilling their official teaching and research responsibilities, they additionally pursue their Ph.D. This differs from structured doctoral programs in other countries where doctoral candidates mainly focus on their own studies rather than simultaneously working as university faculty.

Regarding the first research area mentioned, primarily interview studies have been conducted in which university instructors were asked to elaborate on their teaching-related experiences and in doing so, spontaneously mentioned a variety of emotions. Using this approach, Postareff and Lindblom-Ylänne (2011) found that 92 of 97 university instructors described teaching-related emotions, with enjoyment and enthusiasm being the most frequently mentioned positive emotions, and reluctance being the most frequently mentioned negative emotion. Similarly, Hagenauer and Volet (2014) observed that enjoyment, happiness, and hope were the most frequently mentioned positive emotions compared to annoyance, insecurity, and worry.

Multi-method studies combining interviews and questionnaires have also emerged within the literature. In a study on new faculty members’ emotions, 18 discrete emotions were described throughout interviews inquiring about faculty work experiences (Stupnisky et al., 2016). Following this, survey data indicated that faculty experienced more joy, pride, and boredom for teaching compared to research, and that these emotions played a role in teaching success through perceived value for teaching. Moreover, male faculty were found to experience significantly more anxiety concerning teaching compared to their female counterparts. Using a similar approach, Stupnisky et al. (2019a) found that in pre-tenure faculty, different positive discrete emotions were positively correlated with perceived teaching-related control, value, success, collegiality, and personal balance, while the opposite was found regarding negative emotions, also for teaching-related expectations and professional balance.

Regarding solely quantitative studies, positive teaching emotions (e.g., pride) have been positively associated with student-focused teaching, while negative emotions (e.g., anxiety) have been positively associated with teacher-focused teaching (Trigwell, 2012; see Kordts-Freudinger, 2017 for similar results concerning positive affect). Positive emotions such as joy have also been found to be positively correlated with personal and professional balance, control, value, and perceived success in teaching in early-career faculty, while for negative emotions such as anxiety, the opposite has been documented (Stupnisky et al., 2019b). Thies and Kordts-Freudinger (2019) additionally found that appraisals of value and of self-determination concerning teaching were positively related to positive emotions including enjoyment and pride, whereas appraisals concerning teaching-related time-pressure and control were positively related to negative emotions such as anger and anxiety.

Despite these promising results indicating that emotions play an integral role in university instructors’ professional lives, we are just starting to understand this line of research. Particularly lacking are studies that investigate precursors of emotions in this context from a discrete perspective. By gaining insight into relevant antecedents of university instructors’ discrete emotions, we can achieve a better understanding of how these emotions arise and advance our knowledge of how to foster positive emotions. One theoretically relevant antecedent of emotions is achievement motivation (Pekrun et al., 2006, 2009), where different qualities of achievement motivation, such as achievement goals, may facilitate different achievement emotions.

### Achievement Goals as Antecedents of Discrete Emotions in University Instructors

Discrete emotions have historically been theoretically and empirically intertwined with achievement goals (Pekrun et al., 2006). *Achievement goals* can be defined as the purposes for engaging in competence-related behavior (Elliot and Hulleman, 2017). These goals act as a lens for how one evaluates current and future achievement situations, and underlie different interpretations, behaviors, and reactions including coping and emotion processes (Kaplan and Maehr, 1999; Tuominen-Soini et al., 2008).

Achievement goals are relevant for university instructors as teaching in universities constitutes an achievement context requiring instructors to produce high-quality teaching outcomes, successfully perform under observation, act in a social context, and continuously improve (see Daumiller et al., 2019b). While initial work on achievement goals employed a dichotomous framework including *mastery goals*, which are focused on fostering skills and knowledge, and *performance goals*, which are focused on demonstrating skills and knowledge (Nicholls, 1984; Dweck, 1986; Maehr, 1989; Ames, 1992; see Korn et al., 2019), further differentiations have since been recognized. Fundamentally, an approach (striving to reach certain end states) and an avoidance (striving to avoid certain end states) valence have been established (see Elliot and McGregor, 2001), and mastery and performance goals have been further differentiated based on their content and evaluation standards (e.g., Elliot et al., 2011). Specifically, the mastery goal construct can be further differentiated depending on whether an individual is focused on improvement and self-development, termed *learning goals* in the present work (see Daumiller et al., 2019b), or on mastering the task at hand, termed *task goals*. The general performance goal construct can also be further differentiated depending on whether an individual assesses their competence based on appearing competent, termed *appearance goals*, or on outperforming others, termed *normative goals*. Adding to this, especially in teaching contexts, *work avoidance goals*, which focus on striving to get by with little effort, and *relational goals*, which focus on developing close and caring relationships, have also been regarded as important goal classes (Butler, 2007, 2012; Butler and Shibaz, 2008; Daumiller et al., 2016).

Integrating these distinctions, Daumiller et al. (2019b) proposed an overview model and a respective scale regarding the relevant distinguishable achievement goal classes for describing and analyzing university instructors’ motivations (see Table 1 for the goal classes and example items). Within this, established goal classes from prior frameworks and research were integrated into a comprehensive model suitable for characterizing the full scope of university instructors’ achievement goals (see Daumiller et al., 2019b, for details). Based on this model and the respective scale, a series of studies have been conducted and have proven their suitability for assessing university instructors’ achievement goals as well as their relevance for explaining differences in their experiences and behaviors (e.g., Daumiller et al., 2019a, 2020b; Hein et al., 2019; Janke et al., 2019b; Daumiller and Dresel, 2020a, b). We therefore also adopted this achievement goal model in the present study given prior empirical evidence and its theoretical fittingness.

**TABLE 1:** Overview of the distinguished achievement goals, their sample items, and internal consistencies.

	Goal content	Valence	Sample item	ω_*H*_
			Item stem: “In my current teaching activities…”	
Mastery-based goals	Task	Approach	“… I want to fulfill the different requirements very well.”	0.83
		Avoidance	“… I want to avoid fulfilling the different requirements poorly.”	0.88
	Learning	Approach	“… I want to constantly improve my competences.”	0.89
		Avoidance	“… it is important to me to avoid having my competencies not develop further.”	0.84
Performance-based goals	Appearance	Approach	“… I want to be perceived as competent.”	0.86
		Avoidance	“… I want to avoid being perceived as incompetent.”	0.94
	Normative	Approach	“… I want to be better than my colleagues.”	0.94
		Avoidance	“… I want to avoid being worse than my colleagues.”	0.95
Further goals	Work avoidance	Avoidance	“… I want to have as little to do as possible.”	0.91
	Relational	Approach	“… it is important to me to achieve a personal connection with students.”	0.79

**Reported are the distinguished achievement goals and their definitions based on the model of Daumiller et al. (2019b), as well as their internal consistencies (McDonald’s Omega values).**

Regarding associations between achievement goals and discrete emotions, Pekrun et al. (2006) explained that achievement goals can be “assumed to regulate the achievement-related thoughts and actions that shape […] emotions” (p. 583). They derived clear theoretical expectations about the associations between achievement goals and discrete emotions. In particular, Pekrun et al. (2006, 2009) proposed a model based on the trichotomous framework of achievement goals. Within this model, mastery goals are thought to be centered around the controllability and positive value of achievement activities and outcomes, thereby facilitating increased positive activity emotions such as enjoyment, and decreased negative activity emotions such as boredom or anger. Furthermore, performance approach goals are expected to be focused on achieving success outcomes, the controllability of these outcomes, and their positive value, resulting in increased positive outcome emotions (e.g., pride). Lastly, performance avoidance goals are proposed to be focused on potential failure outcomes, the uncontrollability of these outcomes, and their negative value, facilitating increased negative outcome emotions such as anxiety and shame.

Empirically, these expectations have been strongly supported by findings in student populations. In a study testing the above mentioned theoretical model, Pekrun et al. (2006) reported that mastery goals were positively associated with students’ enjoyment, hope, and pride, and negatively associated with boredom and anger. Additionally, performance approach goals were positively associated with pride, while performance avoidance goals were positively associated with anxiety, hopelessness, and shame. As an extension of these findings, Pekrun et al. (2009) found that mastery goals were positively linked with students’ enjoyment and negatively linked with boredom and anger; performance approach goals were positively linked with pride and hope, and performance avoidance goals were positively linked with anxiety, hopelessness, and shame. Goetz et al. (2016) observed similar relations, with mastery goals having positive associations with enjoyment and negative associations with boredom and anger, performance approach goals having positive associations with pride, and performance avoidance goals having positive associations with anxiety and shame. Similar to the aforementioned findings, a comprehensive meta-analysis including 77 studies documented positive links for students’ mastery approach goals with positive emotions such as enjoyment and hope, as well as positive links for performance avoidance and mastery avoidance goals with negative emotions such as anxiety and anger (Huang, 2011). A review of further empirical evidence linking achievement goals and emotions in students can be found in the work of Goetz et al. (2016).

Aside from the established links between mastery, performance approach, and performance avoidance goals with discrete emotions in students, recent research has also looked into further differentiated goals. Lüftenegger et al. (2016) found that students’ learning-based goals, performance-based goals, and task-approach goals were positively associated with enjoyment. Additionally, task-approach goals were negatively associated with boredom. Studies have also looked into work avoidance goals, finding positive associations with negative affect (King and McInerney, 2014) and boredom (Jarvis and Seifert, 2002).

Concerning studies focused on school teachers and university instructors, to the best of our knowledge, only a mere few exist. In school teachers, mastery and relational goals have been positively related to increased enjoyment, while work avoidance goals have been related to reduced enjoyment and increased anxiety and anger (Wang et al., 2016). Janke et al. (2019a) found similar results in school teachers with mastery goals being positively related to enjoyment, performance approach goals being negatively related to anxiety, and performance avoidance as well as work avoidance goals being positively related to anxiety and negatively related to enjoyment. With respect to university instructors, task approach, normative approach, and relational goals have been positively related to positive affect, and normative avoidance and work avoidance goals have been negatively related to positive affect (Daumiller et al., 2019b). While the latter study is a promising stepping stone—having been the first to look into achievement goals and emotional experiences of university instructors—further studies are necessary, especially concerning discrete emotions, which have yet to be examined in this population. Moreover, including and beyond the population of university instructors, more research is needed concerning the achievement goal–emotion link past the trichotomous model, as also suggested by Pekrun et al. (2006, 2009), Daniels et al. (2009), and Goetz et al. (2016). Our study aims to address these research gaps by examining achievement goals on a differentiated level with discrete emotions, and by investigating whether prior findings primarily based on student and school teacher populations can be generalized to university instructors.

### Achievement Goals as Moderators of the Relationship Between Job Demands and Emotions

University instructors often experience high job demands (see special issue of Kinman and Johnson, 2019) and, in turn, may face consequences such as burnout (Lackritz, 2004) or negative work-related emotions (Mudrak et al., 2018). At the same time, there is individual variation in these associations. This is also reflected in instructors reporting high job satisfaction despite simultaneously having high job stress (Shin and Jung, 2014). To gain insight into this variation, identifying potential moderators constitutes a promising avenue. As achievement goals shape perceptions of achievement situations and underlie interpretations, behaviors, and reactions including coping and emotion (Kaplan and Maehr, 1999; Tuominen-Soini et al., 2008), it is theoretically plausible that stronger or weaker relationships may occur between university instructors’ job demands and discrete emotions depending on the types of goals they pursue.

Concerning individual job demands, given that time, resource, and workload constraints have been highlighted as central stressors in the higher education context (Kinman and Jones, 2008), we were especially interested in the discrepancy between the ideal amount of time that university instructors would like to spend on their teaching activities, compared to the actual amount of time that they spend on them, labeled as their *teaching-related task discrepancy*. Within this, we expect learning approach and work avoidance goals to act as moderators based on their respectively adaptive and maladaptive nature for university instructors’ work experiences (see Daumiller et al., 2016, 2019b). For instructors who strongly pursue learning approach goals and are focused on developing knowledge and skills, job demands may be perceived as learning opportunities and facilitate more adaptive associations with emotions (i.e., a buffer effect in the form of primary appraisal; see Daumiller and Dresel, 2020b, for similar argumentation). In contrast, those who pursue work avoidance goals and are focused on getting by with little effort may not perceive or handle job demands in a productive manner and rather use these goals as coping mechanisms, perpetuating maladaptive relations with emotions (negative primary and secondary appraisals; see Folkman et al., 1986). Thus, how strongly learning approach and work avoidance goals are pursued may alter the way in which university instructors’ job demands are associated with their emotions on the basis of their interpretation and handling of job demands.

### Current Study and Hypotheses

The relationship between university instructors’ achievement goals and discrete emotions is a theoretically promising yet largely untapped research avenue. Moreover, achievement goals may constitute important moderators to explain variation in how university instructors emotionally respond to job demands. The aims of the present research were therefore to examine the link between university instructors’ achievement goals and discrete emotions, and, additionally, to examine whether learning approach and work avoidance goals moderate the relationship between job demands and discrete emotions. To ensure that the observed relations were robust, we controlled for age, academic rank, and gender.

Building on prior evidence on the relations between achievement goals and discrete emotions, we tested the following hypotheses:

(H1) Mastery approach goals (i.e., learning and task approach goals) are positively associated with enjoyment, and negatively associated with boredom and anger.
(H2) Mastery avoidance goals (i.e., learning and task avoidance goals) are positively associated with boredom, anxiety, and anger.
(H3) Performance approach goals (i.e., normative and appearance approach goals) are positively associated with pride.
(H4) Performance avoidance goals (i.e., normative and appearance avoidance goals) are positively associated with anxiety and shame.
(H5) Relational goals are positively associated with enjoyment.
(H6) Work avoidance goals are positively associated with anxiety, boredom, and shame.

**FIGURE 1:**
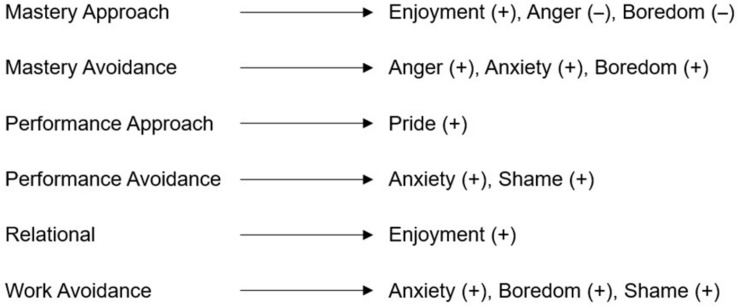
Expected direct associations between achievement goals and discrete emotions. Modified visual based on Pekrun et al. (2009).

Research suggests that task and learning components of mastery goals as well as appearance and normative components of performance goals may be differentially associated with university instructors’ professional experiences (see Daumiller et al., 2019b). At the same time, there is little research indicating how exactly they may differ in terms of discrete emotions. We therefore examined differences between their associations without directed hypotheses. A comprehensive overview of these hypotheses can be found in Figure 1.

**FIGURE 2:**
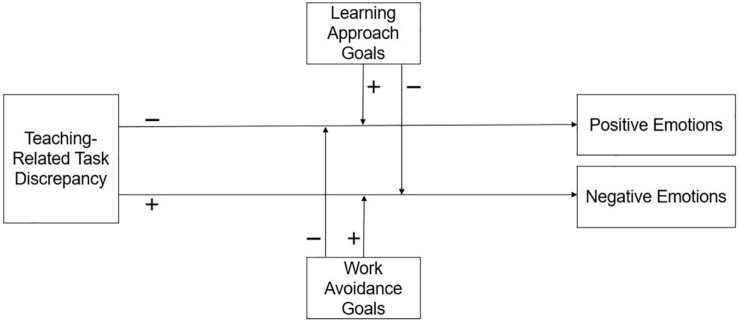
Proposed moderation hypotheses: Learning approach goals have a positive effect on the negative relation between teaching-related task discrepancy and positive emotions, and a negative effect on the positive relation between teaching-related task discrepancy and negative emotions. Work avoidance goals have a positive effect on the positive relation between teaching-related task discrepancy and negative emotions, and a negative effect on the negative relation between teaching-related task discrepancy and positive emotions.

Based on the theoretical nature of learning approach and work avoidance goals and how they may be associated with different interpretations of stressors, and in turn, emotional experiences (as depicted in Figure 2), we expected:

(H7) Learning approach and work avoidance goals moderate the relationship between teaching-related task discrepancy and emotions: The stronger the learning approach goals, the more positive the relations between task discrepancy and positive emotions, and the more negative the relations between task discrepancy and negative emotions (i.e., learning approach goals encourage adaptive relations with emotions). The stronger the work avoidance goals, the more positive the relations between task discrepancy and negative emotions, and the more negative the relations between task discrepancy and positive emotions (i.e., work avoidance goals exacerbate maladaptive relations with emotions).

## Materials and Methods

### Procedure and Sample

To test the proposed hypotheses, 439 university instructors (18.45% full professors, 46.47% academic staff with Ph.D., 35.08% academic staff without Ph.D.) employed at 18 universities within Germany and Austria participated in an online survey. Only those with teaching responsibilities were eligible to participate. Participants were incentivized with a 5 € voucher or donation to a charity for their time. The sample included 205 females, 230 males, and 4 individuals not wanting to disclose their gender, with an average age of 38.44 years (*SD*=10.10). Years of teaching ranged from 1 to 42 (*M*=8.56, *SD*=8.12). The data used in the current study were retrieved on October 31, 2019, and marked that of the first time point of a larger longitudinal study (Authors anonymized, 2019).

### Measurements

#### Achievement Goals for Teaching

Achievement goals for teaching were measured using the scale by Daumiller et al. (2019b). Following the item stem “In my current teaching activities,” four questions were asked for each goal class, which are described along with sample items in Table 1. Reliability analysis of each goal category indicated excellent internal consistency (see Omega values in Table 1). Answers were recorded on a Likert-type scale ranging from 1 (*do not agree at all*) to 8 (*agree completely*).

#### Discrete Emotions

We measured university instructors’ enjoyment, pride, anger, anxiety, shame, and boredom using the single item scale developed in the study of Goetz et al. (2016). When asked about how often they experienced the aforementioned emotions concerning their work as a university instructor in the past month, the participants rated each emotion using a five-point Likert-type scale ranging from 1 (*not at all*) to 5 (*very often*).

#### Teaching-Related Task Discrepancy

To measure teaching-related task discrepancy, we asked instructors to indicate the percentage of time that they currently spend on teaching-related tasks and the percentage of time that they would ideally like to spend on teaching-related tasks. We then calculated the deviation between the ideal percentage of time and the actual percentage of time. Thus, an instructors’ teaching-related task discrepancy could theoretically be any value ranging from 0 to 100%. Low values indicate alignment between ideal and current time allocation, thus representing a low discrepancy. High values indicate that instructors either spent more or less time on teaching-related tasks than desired, representing a high discrepancy. This reflects our understanding that the psychological mechanisms leading to dissatisfaction and emotional experiences should primarily be a function of how aligned the time allocation is with instructors’ desires. Thus, spending more or spending less time than desired on teaching-related duties may be dissatisfying.^1^

### Analyses

#### Multiple Regression Analyses

To test our hypotheses on the relations between achievement goals and discrete emotions, multiple regression analyses were conducted with Mplus (Muthén and Muthén, 2017). The robust weighted least squares (WLSMV) estimator was used to estimate the model parameters as the emotions were measured as single items with five categories. We additionally allowed for associations between predictors. Separate regressions were computed for each discrete emotion with all achievement goals as predictors. The influence of age, academic rank, and gender was controlled for in these analyses.

#### Moderation Analyses

Moderation analyses were additionally conducted with Mplus (Muthén and Muthén, 2017). We tested whether the relationship between teaching-related task discrepancy and a given discrete emotion changed depending on the strength to which learning approach and work avoidance goals were pursued. In all analyses, emotions were predicted from teaching-related task discrepancy, learning or work avoidance goals, and the interaction between task discrepancy and the respective goal. We standardized all variables prior to analyses and calculated the interaction terms by multiplying teaching-related task discrepancy with either learning approach or work avoidance goals (Cohen et al., 2003). Again, age, academic rank, and gender were controlled for.

## Results

### Descriptive Statistics and Intercorrelations

Descriptive statistics (see Table 2) revealed moderate to high means for the achievement goals with the exception of work avoidance goals. Moreover, relatively large variances were observed, implying considerable inter-individual differences. Similar trends were found for the different emotions, with the exception of anxiety, shame, and boredom, which had slightly lower mean values compared to the other emotions. Concerning teaching-related task discrepancy, the mean percentage was 12% with a notably high variance. About one third of the participants (34.6%) wished to spend a lower percentage of their time on teaching-related activities, others (18.5%) reported no discrepancy at all, and some (44.6%) wished to spend a larger percentage of their time on teaching-related activities. Moreover, all variables had mostly weak to moderate correlations with one another, with correlations between goals and emotions having theoretically sensible links.

**TABLE 2:** Descriptive statistics and correlations of achievement goals, emotions, and task discrepancy.

	Descriptive statistics			Bivariate correlations															
	*M*	*SD*	Skew	1	2	3	4	5	6	7	8	9	10	11	12	13	14	15	16
Achievement goals																			
[1] Learning approach	6.96	1.14	–1.66																
[2] Learning avoidance	6.13	1.76	–0.91	**0.54**															
[3] Task approach	7.25	0.85	–1.61	**0.55**	**0.40**														
[4] Task avoidance	7.02	1.42	–2.02	**0.29**	**0.51**	**0.44**													
[5] Appearance approach	6.02	1.48	–0.78	**0.23**	**0.20**	**0.33**	**0.20**												
[6] Appearance avoidance	6.07	1.90	–0.97	0.09	**0.26**	**0.24**	**0.48**	**0.60**											
[7] Normative approach	3.84	1.99	0.07	–0.08	0.04	0.03	**0.11**	**0.50**	**0.44**										
[8] Normative avoidance	5.48	2.23	–0.67	**0.11**	**0.24**	**0.22**	**0.43**	**0.48**	**0.71**	**0.53**									
[9] Relational	5.11	1.59	–0.24	**0.17**	**0.19**	**0.18**	**0.15**	**0.24**	**0.23**	**0.26**	**0.16**								
[10] Work avoidance	2.85	1.78	0.82	−**0.29**	−**0.19**	−**0.35**	−**0.13**	**0.09**	**0.14**	**0.28**	**0.18**	0.05							
Discrete emotions																			
[11] Enjoyment	3.97	0.75	–0.45	**0.18**	**0.11**	**0.19**	**0.11**	0.05	0.06	< 0.01	0.07	0.08	−**0.16**						
[12] Pride	3.32	0.87	–0.27	**0.10**	**0.12**	**0.13**	**0.11**	**0.18**	**0.11**	0.09	0.07	**0.10**	–0.03	**0.41**					
[13] Anger	2.76	0.97	0.23	–0.10	0.02	–0.09	–0.02	–0.02	–0.05	0.01	–0.03	–0.05	–0.02	−**0.25**	−**0.16**				
[14] Anxiety	1.95	0.99	0.90	0.07	0.05	0.04	< 0.01	**0.12**	**0.13**	0.07	0.07	0.07	0.09	−**0.14**	–0.05	**0.22**			
[15] Shame	1.56	0.81	1.34	0.01	–0.02	–0.01	< 0.01	**0.10**	0.13	0.04	0.05	**0.12**	**0.12**	−**0.18**	−**0.10**	**0.21**	**0.41**		
[16] Boredom	1.77	0.91	1.13	−**0.16**	−**0.11**	**0.13**	−**0.11**	–0.03	–0.03	0.05	–0.05	0.06	**0.21**	−**0.24**	−**0.14**	**0.14**	**0.10**	**0.19**	
Task discrepancy	12.00	11.65	1.47	0.03	0.02	**0.10**	–0.03	0.05	0.02	–0.02	0.01	–0.02	0.01	–0.09	–0.05	0.08	**0.11**	0.04	−**0.01**
Control variables																			
Age	38.44	10.10	0.78	–0.01	< 0.01	–0.05	0.04	−**0.10**	–0.08	–0.04	< 0.01	0.07	−**0.16**	**0.11**	–0.02	0.06	−**0.21**	−**0.11**	−**0.23**
Full professor (1 = yes, 0 = no)	0.18	0.39	1.63	–0.08	–0.03	–0.10	0.04	–0.09	–0.04	0.05	< 0.01	0.02	0.04	0.07	0.01	0.08	–0.06	–0.07	−**0.13**
Ph.D. (1 = yes, 0 = no)	0.35	0.48	0.63	< 0.01	–0.02	0.05	–0.07	0.04	0.03	–0.01	0.01	< 0.01	**0.12**	–0.09	–0.01	–0.02	**0.20**	0.09	**0.17**
Gender (1 = ♂, 2 = ♀)	1.49	0.52	0.26	**0.18**	0.09	**0.15**	0.01	**0.11**	0.04	−**0.15**	0.01	0.02	−**0.12**	0.07	0.05	–0.04	**0.16**	0.05	–0.11

**For (ordinal-scaled) emotions, Spearman’s correlations are presented; otherwise, Pearson’s correlations. Theoretical range for achievement goals: 1–8, emotions: 1–5, and task discrepancy: 0–100%. Statistically significant correlations (p < 0.05) are displayed in boldface.**

### Associations Between Achievement Goals and Discrete Emotions

Regarding the associations between the achievement goals and discrete emotions, a number of differential relations were found in the structural equation models. See Table 3 for the corresponding values.

**TABLE 3:** Multiple regression analyses for achievement goals as predictors of emotions.

	Model 1: Enjoyment	Model 2: Pride	Model 3: Anger	Model 4: Anxiety	Model 5: Shame	Model 6: Boredom
Achievement goals						
Learning approach	** 0.11 (0.06)**	0.09 (0.07)	**−0.18 (0.07)**	0.08 (0.07)	0.05 (0.08)	** −0.16 (0.07)**
Learning avoidance	−0.05(0.06)	0.01 (0.07)	** 0.17 (0.06)**	0.05 (0.07)	−0.08(0.08)	0.04 (0.07)
Task approach	0.10 (0.06)	0.06 (0.07)	−0.07(0.07)	−0.05(0.07)	−0.05(0.07)	−0.03(0.07)
Task avoidance	0.04 (0.06)	0.03 (0.08)	−0.05(0.07)	−0.07(0.06)	0.04 (0.08)	−0.04(0.07)
Appearance approach	−0.07(0.06)	** 0.13 (0.06)**	0.06 (0.06)	−0.02(0.07)	0.02 (0.08)	−0.01(0.07)
Appearance avoidance	0.05 (0.07)	0.02 (0.07)	−0.09(0.08)	** 0.21 (0.09)**	** 0.24 (0.10)**	−0.06(0.09)
Normative approach	0.01 (0.07)	0.05 (0.06)	0.04 (0.06)	0.05 (0.06)	−0.07(0.07)	0.03 (0.07)
Normative avoidance	0.05 (0.08)	−0.07(0.08)	0.03 (0.07)	−0.08(0.08)	−0.11(0.09)	−0.03(0.09)
Relational	0.05 (0.05)	0.04 (0.05)	−0.02(0.06)	0.04 (0.05)	** 0.13 (0.05)**	**0.10 (0.05)**
Work avoidance	**−0.13 (0.06)**	−0.02(0.06)	−0.08(0.06)	0.03 (0.06)	** 0.11 (0.06)**	**0.15 (0.05)**
Control variables						
Age	0.08 (0.08)	−0.01(0.06)	−0.06(0.07)	**−0.20 (0.08)**	−0.10(0.08)	** −0.20 (0.07)**
Full professor (1 = yes, 0 = no)	0.05 (0.06)	0.04 (0.06)	** 0.10 (0.06)**	0.11 (0.07)	−0.02(0.07)	−0.07(0.07)
Ph.D. (1 = yes, 0 = no)	−0.03(0.07)	< 0.01(0.06)	0.01 (0.06)	0.14 (0.06)	0.02 (0.07)	0.05 (0.06)
Gender (1 = ♂, 2 = ♀)	0.06 (0.05)	0.03 (0.05)	−0.01(0.05)	** 0.17 (0.05)**	0.04 (0.06)	** −0.10 (0.05)**
*R*^2^	0.09	0.06	0.05	0.15	0.10	0.17

**Reported are the standardized regression coefficients with their standard errors in parentheses. Running the model without age, academic rank, and gender as controls yielded no significant differences in parameter estimates. Statistically significant coefficients (p < 0.05) are displayed in boldface. All models were fully saturated and yielded a perfect fit to the data.**

We found enjoyment to be positively associated with learning approach goals and negatively associated with work avoidance goals. Conversely, pride was only associated with appearance approach goals: instructors focused on wanting to make a good impression reported stronger pride than those less in pursuit of these goals.

For the negative emotions, we found anger to be negatively associated with learning approach goals and positively associated with learning avoidance goals, while no such associations were found for anxiety and shame. Instead, we found a moderate positive association between instructors’ pursuit of appearance avoidance goals and their experiences of anxiety and shame. Additionally, relational and work avoidance goals were positively associated with the experience of shame.

Finally, we found similar trends for boredom: positive associations with work avoidance and relational goals and a negative association with learning approach goals.

Altogether, achievement goals explained up to 17% of the variance in emotions. Additional analyses, also without the control variables, and comparisons with bivariate correlations spoke to the robustness of these results and did not provide indication of suppressor effects.

### Achievement Goals as Moderators Between Task Discrepancy and Emotions

Concerning the role of learning approach and work avoidance goals as moderators in the relationship between teaching-related task discrepancy and discrete emotions (see Table 4), we did not find consistent interaction effects. Contrary to expectations, we found that instructors with high task discrepancy reported rather similar levels of pride irrespective of the strength of their learning goals (see Supplementary Figure S1). In comparison, instructors with low task discrepancy reported more pride when having strong learning goals, and less pride when combined with weak learning goals (i.e., negative interaction). For pride and work avoidance goals, in line with our expectations, we found that instructors with stronger work avoidance goals and more task discrepancy experienced less pride, and that there was positive interaction between both. However, closer inspection of the simple slope plots (see Supplementary Figure S2) revealed that low task discrepancy and low work avoidance goals was associated with more pride, while instructors with strong work avoidance goals or high task discrepancy did not differ significantly from each other.

**TABLE 4:** Moderation of the associations between task discrepancy and emotions by learning approach and work avoidance goals.

	Model 1: Enjoyment	Model 2: Pride	Model 3: Anger	Model 4: Anxiety	Model 5: Shame	Model 6: Boredom
**Learning approach goal models**						
Learning approach	**0.27 (0.08)**	**0.28 (0.08)**	**−0.17 (0.07)**	0.06 (0.07)	−0.05(0.08)	**−0.27 (0.07)**
Task discrepancy	0.46 (0.36)	**0.59 (0.33)**	−0.34(0.35)	0.12 (0.26)	−0.10(0.31)	−0.44(0.29)
Interaction	−0.56(0.37)	**−0.65 (0.33)**	0.42 (0.34)	−0.02(0.26)	0.17 (0.32)	0.45 (0.29)
Control variables						
Age	**0.12 (0.07)**	−0.01(0.07)	< 0.01(0.05)	**−0.18 (0.08)**	−0.12(0.09)	**−0.21 (0.07)**
Full professor (1 = yes, 0 = no)	0.03 (0.07)	0.04 (0.06)	0.08 (0.06)	0.10 (0.07)	< 0.01(0.07)	−0.06(0.07)
Ph.D. (1 = yes, 0 = no)	−0.03(0.06)	< 0.01(0.06)	0.01 (0.06)	**0.14 (0.06)**	0.04 (0.07)	0.08 (0.06)
Gender (1 = ♂, 2 = ♀)	0.08 (0.06)	0.03 (0.06)	−0.01(0.05)	**0.18 (0.05)**	0.05 (0.06)	**0.13 (0.05)**

**Work avoidance goal models**						

Work avoidance	**−0.19 (0.08)**	**−0.15 (0.07)**	0.07 (0.07)	< 0.01(0.07)	0.04 (0.08)	**0.16 (0.07)**
Task discrepancy	−0.12(0.11)	**−0.23 (0.10)**	**0.22 (0.09)**	0.02 (0.09)	−0.12(0.12)	−0.09(0.10)
Interaction	0.07 (0.13)	**0.24 (0.11)**	**−0.20 (0.10)**	0.12 (0.09)	**0.22 (0.12)**	0.09 (0.11)
Control variables						
Age	0.08 (0.07)	−0.03(0.07)	< 0.01(0.06)	**−0.17 (0.08)**	−0.09(0.09)	**−0.17 (0.07)**
Full professor (1 = yes, 0 = no)	0.04 (0.07)	0.03 (0.06)	0.09 (0.06)	0.09 (0.07)	−0.03(0.07)	−0.07(0.07)
Ph.D. (1 = yes, 0 = no)	−0.02(0.06)	< 0.01(0.06)	< 0.01(0.06)	**0.14 (0.06)**	0.03 (0.07)	0.07 (0.06)
Gender (1 = ♂, 2 = ♀)	0.08 (0.05)	0.04 (0.05)	−0.02(0.05)	**0.20 (0.05)**	0.07 (0.06)	**−0.12 (0.05)**

**Reported are the standardized coefficients with their standard errors in parentheses of the individual moderation analyses concerning teaching-related task discrepancy and emotions moderated by achievement goals. Statistically significant effects (p < 0.05) are displayed in boldface. All models were fully saturated and yielded a perfect fit to the data.**

Furthermore, our results indicated that instructors with higher task discrepancy reported more anger, however, in contrast to our expectations, this was negatively moderated by the strength of their work avoidance goals—with low work avoidance goals in combination with high task discrepancy being associated with more anger (see Supplementary Figure S3). Finally, for experiences of shame, we did not find any main effects for work avoidance goals or task discrepancy, however, as expected, there was a positive interaction, meaning that high task discrepancy combined with strong work avoidance goals was associated with particularly high levels of shame (crossover interaction; see Supplementary Figure S4).

## Discussion

Achievement goals have been ascribed an important role for predicting discrete achievement emotions in students (for an overview, see Huang, 2011; Linnenbrink-Garcia and Barger, 2014) and school teachers (Wang et al., 2016; Janke et al., 2019a). Although achievement goals have been found to be associated with university instructors’ positive affect (Daumiller et al., 2019b), to the best of our knowledge, our study is the first to examine associations with discrete emotions. Generally, the positive and negative emotional experiences of instructors can be considered fundamental for their overall subjective well-being, as well as the learning experiences of their students through their teaching behaviors (see Frenzel et al., 2016; Mendzheritskaya and Hansen, 2019). However, each discrete emotion exhibits distinct qualitative features and can have different antecedents and effects (Lazarus, 2006). Thus, studying how individual goals relate to discrete emotions is an important avenue to allow for a fine-grained view of these links. Beyond gaining further evidence that positive and negative emotional experiences are respectively beneficial or maladaptive for instructors, it is also important to understand how and why these experiences occur. Moreover, to further understand individual differences in instructors’ emotional experiences, we additionally investigated learning approach and work avoidance goals as moderators between job demands and discrete emotions.

An important strength of the present study is that we examined achievement goals and emotions in the understudied and at-risk population of university instructors. Adding to this, we considered achievement goals in a differentiated manner and took a discrete approach on emotions, allowing for a comprehensive and detailed understanding of their associations. Finally, our study afforded first insights into the possible role of achievement goals as moderators between job demands and emotions. In general, our results suggest that achievement goals are important motivational forces associated with university instructors’ discrete emotions.

### Insights Into Associations Between Achievement Goals and Discrete Emotions

Regarding mastery approach goals, the findings from our regression analyses partially supported Hypothesis 1. Largely consistent with prior research (e.g., Pekrun et al., 2006, 2009; Huang, 2011; Goetz et al., 2016), we found that learning approach goals had positive associations with enjoyment, as well as negative associations with anger and boredom. As both increased enjoyment as well as the endorsement of learning approach goals can be considered adaptive for instructors’ well-being, research efforts should be made to further understand how they can be feasibly fostered while considering relevant factors such as particularly demanding work conditions. On the other hand, this adaptive pattern was not found for task approach goals, in contrast to the findings of Daumiller et al. (2019b), which suggested that task approach goals may be even more advantageous for university instructors’ experiences of positive affect than learning approach goals. At the same time, although related, affective experiences do not equate to discrete emotions, and thus, differences in these finer relations can be expected. Moreover, the differential relations that emerged for learning approach and task approach goals with emotions highlight the importance of taking a comprehensive approach to investigating these links, which should be followed up on in future research.

Concerning mastery avoidance goals, the findings of our regression analyses were partially in support of Hypothesis 2 in that learning avoidance goals were positively associated with anger. This is consistent with prior findings such as those of Huang (2011), who found mastery avoidance goals to have large correlations with negative achievement emotions. This may indicate that instructors who are struggling to avoid losing or not developing their competencies may become frustrated with their work, potentially eventuating in anger. Nevertheless, as research shows that university instructors may be susceptible to negative emotional experiences, further studies should be conducted to determine the severity and persistence of this association. Moreover, we did not find statistically significant associations with task avoidance goals, again speaking to the importance of further differentiating mastery avoidance goals into both learning avoidance and task avoidance components.

For performance approach and avoidance goals, our findings partially supported Hypothesis 3 and Hypothesis 4. Appearance approach goals were positively linked with pride, while appearance avoidance goals were positively linked with anxiety and shame. This pattern of results has been consistently found in studies examining the general construct of performance avoidance goals with emotions (e.g., Pekrun et al., 2009; Goetz et al., 2016). On the one hand, high levels of pride can be considered beneficial, as this emotion implies that instructors feel that they are doing a good job. On the other hand, when pride is connected with appearance goals as in the present study, this may be less beneficial and rather suggest that university instructors’ feelings of self-praise depend on how they are perceived by others. Longitudinal research should be conducted to determine how these associations impact university instructors’ well-being and work satisfaction over a longer period of time. Opposed to appearance goals, no statistically significant associations were found for normative approach or normative avoidance goals with discrete emotions. This may indicate that in the context of higher education teaching, appearing competent in front of others may be especially relevant for university instructors’ emotions, while outperforming others (normative strivings) may be less so.

In terms of university instructors’ relational goals, in contrast to Hypothesis 5 and prior findings (e.g., Wang et al., 2016), regression analyses revealed positive associations with shame and boredom. A possible explanation for this finding may be that it is likely difficult to foster close and caring relationships with students in the context of higher education. Here, classes typically have many students, personal interactions are limited, and teacher-focused instruction styles are more common. Moreover, not all personal interactions between university instructors and students are positive and in turn, do not always lead to positive outcomes. Following this interpretation, it could be the case that when university instructors attempt to foster these relationships as an important personal goal but are unsuccessful due to personal interactions being limited in higher education teaching, this may lead to feelings of shame concerning their lack of success, as well as boredom regarding not being able to fulfill their personal interests. If future research confirms this unexpected finding, important implications could be derived, not only for university instructors and their own emotions, but also in terms of fostering a positive environment with their students including beneficial interactions and relationships (see Hagenauer and Volet, 2014). Specifically, researchers may consider investigating how university instructors monitor and pursue relational goals, including student reports on perceptions of close and caring teacher-student interactions.

Concerning work avoidance goals, Hypothesis 6 was supported in that positive associations were found with boredom and shame in our regression analyses (see Jarvis and Seifert, 2002; King and McInerney, 2014, for similar results). Additionally, work avoidance goals were negatively related to enjoyment, though we did not find statistically significant associations with anxiety. It is plausible that attempting to reduce workload by means of putting forth as little effort as possible may ultimately lead to feelings of shame, boredom, and reduced enjoyment, all of which are detrimental to instructors’ well-being. Although there is minimal empirical evidence surrounding work avoidance goals and discrete emotions, their maladaptive nature has been suggested in other studies in the university instructor context (e.g., Daumiller et al., 2016, 2019b). Thus, this goal type can be marked as particularly maladaptive and should be further examined as a potential risk factor. Further research should be conducted to examine if the maladaptive link between work avoidance goals and emotions impacts other facets of university instructors’ work lives.

Taken together, the associations between achievement goals and emotions were statistically significant, with achievement goals explaining between 5 and 17% of the variance in emotions. This falls in the expected range, as apart from achievement goals, emotions are influenced by a number of other variables. Consequently, as noted by Pekrun et al. (2006), it is likely not “reasonable to expect goals to explain all or even most of the variance” (p. 595). Moreover, we did not observe that controlling for age, academic rank, or gender altered the associations found in the present study. Regarding age and academic rank, when interpreting these associations, it should be borne in mind that, as previously mentioned, Ph.D. students in the present study were regular university employees, and therefore may be accustomed to teaching responsibilities similar to their older and higher-ranking counterparts. Adding to this, emotions were not found to differ depending on gender, although some studies indicate that gender differences exist regarding university instructors’ emotions (Stupnisky et al., 2016) as well as variables similar and related to emotions such as stress (e.g., O’Laughlin and Bischoff, 2005; Hart and Cress, 2008).

In terms of theory-driven advances, aside from those already mentioned, there are a number of suggestions that can be drawn from the present research. Our findings indicate that the relations found between goals and emotions in student and school teacher populations are comparable to those found in university instructors, especially regarding the respectively adaptive and maladaptive links between mastery and work avoidance goals. The unique links found between the further specified mastery goal class (i.e., learning and task goals) and performance goal class (i.e., appearance and normative goals) with emotions imply that taking a finer approach to researching this topic matters and can lend important qualitative information that may otherwise remain undetected. Lastly, the incorporation of this goal–emotion link into other relevant lines of research for university instructors’ teaching experiences, such as control and value appraisals teaching styles or perceived success should be followed up on.

### Learning Approach and Work Avoidance Goals as Moderators

We hypothesized that learning approach and work avoidance goals would act as moderators between job demands and emotions (Hypothesis 7). However, we did not find consistent findings to support our hypothesis. In particular, we expected that the stronger the instructors’ learning approach goals were, the more favorably they would interpret job demands, resulting in more adaptive relations between task discrepancy and emotions. In contrast, we found that strong learning goals paired with low instead of high task discrepancy was associated with the highest levels of pride, meaning that for individuals with strong learning goals, higher task discrepancy was actually associated with less pride. This unexpected finding may be explained by the experience of pride possibly being tied not only to goal content but also to whether one manages to achieve one’s goals (see Tracy and Robins, 2004), which might be particularly difficult when faced with high task discrepancy. In other words, if an instructor does not have as much time for teaching-related tasks as they desire, they may not have enough opportunities to reach their goals.

Besides this, we expected that instructors with strong work avoidance goals would react unfavorably to stressors, promoting maladaptive associations between their task discrepancy and emotions. Our results provided indications of this assumption regarding shame, however, for anger and pride, we found contrasting results: Instead of amplifying, work avoidance goals mitigated the positive association between task discrepancy and experiences of anger and the negative association between task discrepancy and pride. One potential suggestion for these inconsistent findings could be that the pursuit of strong work avoidance goals may act as a maladaptive coping mechanism providing short-term relief by avoiding work rather than addressing it to alleviate long-term stress. In consequence, instructors who strongly seek to keep their workload low as a response to high task discrepancy may initially feel more positive or rather indifferent in terms of their emotions (possibly indicated by more pride and less anger as observed in the present study), which over time may eventuate in negative emotional experiences such as shame.

Nevertheless, these moderation findings should solely be considered as encouragement for further research as they were rather inconsistent. On this note, it is important to consider that the modeled associations between goals and emotions reflect not only the influence that goals exert on emotions, but also the potential influence that emotions have on goals. This may have impacted our findings, as it could be the case that teaching-related task discrepancy influences the statistical effect from goals on emotions, but that the statistical effects from emotions on goals are not influenced by task discrepancy. As both directions are possible with our cross-sectional design, the moderation effects therefore may be more difficult to detect. Adding to this, it is possible that the measure that we used to assess job demands (i.e., teaching-related task discrepancy) did not fully capture university instructors’ job demands, which encompass stressors beyond work time allocation, including their emotional burden and work conditions. Future research that incorporates more elaborate measures such as occupational stressors should be conducted.

### Limitations and Future Directions

Despite the strengths of the present research, a number of limitations need to also be acknowledged. First, as the study design was correlational, causality cannot be determined. Thus, while achievement goals may have influenced emotions in accordance with prior literature, it may also be the case that emotions influenced goals, that a reciprocal causation is present, that other variables influenced these associations, or any combination of these possibilities. As previously discussed, this may be particularly relevant for explaining the findings of the moderation analyses. Future research should employ experimental and longitudinal designs to understand temporal effects. Second, while we focused on the teaching domain, examining differences in the associations between achievement goals and discrete emotions simultaneously in other domains such as research also constitutes an important avenue. In line with this, examining job demands tied to other responsibilities such as research or administrative tasks should also be considered. Next, our measures were not perfectly symmetrical in the time frames that they referred to when asking participants to complete the items. Specifically, participants were asked to refer to their emotions experienced “in the past month,” while for achievement goals and task discrepancy, they were asked to refer to the “current teaching situation.” Given this lack of symmetry, the current findings may be considered a conservative estimate on the relations between achievement goals and emotions, and this point should be considered in future research. Additionally, to measure emotions in the current study, we used a validated measure including emotions as single items, however, future studies might consider using more in-depth measures per emotion to gather further information. On a similar note, given that we relied on self-report measures for all variables, which although are typically suitable for assessing subjective experiences (see Pekrun, 2020), single-source bias cannot be ruled out. Future studies should implement relevant control measures to detect such biases, such as social desirability.

## Conclusion

In sum, the results from the current study are encouraging and allow us to conclude that university instructors’ achievement goals are important for better understanding the discrete emotions that they experience. Learning approach goals appear to be particularly adaptive for their emotions, while work avoidance goals seem especially maladaptive. Adding to this, unique associations were found regarding further differentiated goals, supporting the point of conceptualizing achievement goals on a more fine-grained level when assessing university instructors’ emotions. This study should act as a stepping stone for future researchers to expand on in terms of understanding causality and temporal trends and incorporating the goal–emotion link as a strategy to foster adaptive achievement goals and positive work-related emotions.

## Data Availability Statement

The datasets generated for this study are available on request to the corresponding author.

## Ethics Statement

Ethical review and approval was not required for the study on human participants in accordance with the local legislation and institutional requirements. The participants provided their informed consent to participate in this study.

## Author Contributions

All authors listed have made a substantial, direct and intellectual contribution to the work, and approved it for publication.

## Conflict of Interest

The authors declare that the research was conducted in the absence of any commercial or financial relationships that could be construed as a potential conflict of interest.

## Footnotes

1 We acknowledged that despite this theoretical premise, the associations with teaching-related task discrepancy may differ based on whether instructors spend more or less time on teaching-related tasks than they would like. Therefore, we conducted a set of ancillary analyses where we also controlled for the direction of task discrepancy. We did not find the results to change depending on the direction of task discrepancy, which affirms our notion of primarily the magnitude of task discrepancy, rather than the valence, mattering for the psychological processes of interest in the present work.
