# Supplementary material for: Exploring University Instructors’ Achievement Goals and Discrete Emotions
Source: Front Psychol. 2020 Aug 7;11:1484. doi: 10.3389/fpsyg.2020.01484 (PMC7426512; doi:10.3389/fpsyg.2020.01484)
Supplement: Supplementary file 1 [file Data_Sheet_1.pdf]

Supplementary Material

1. Supplementary Figures

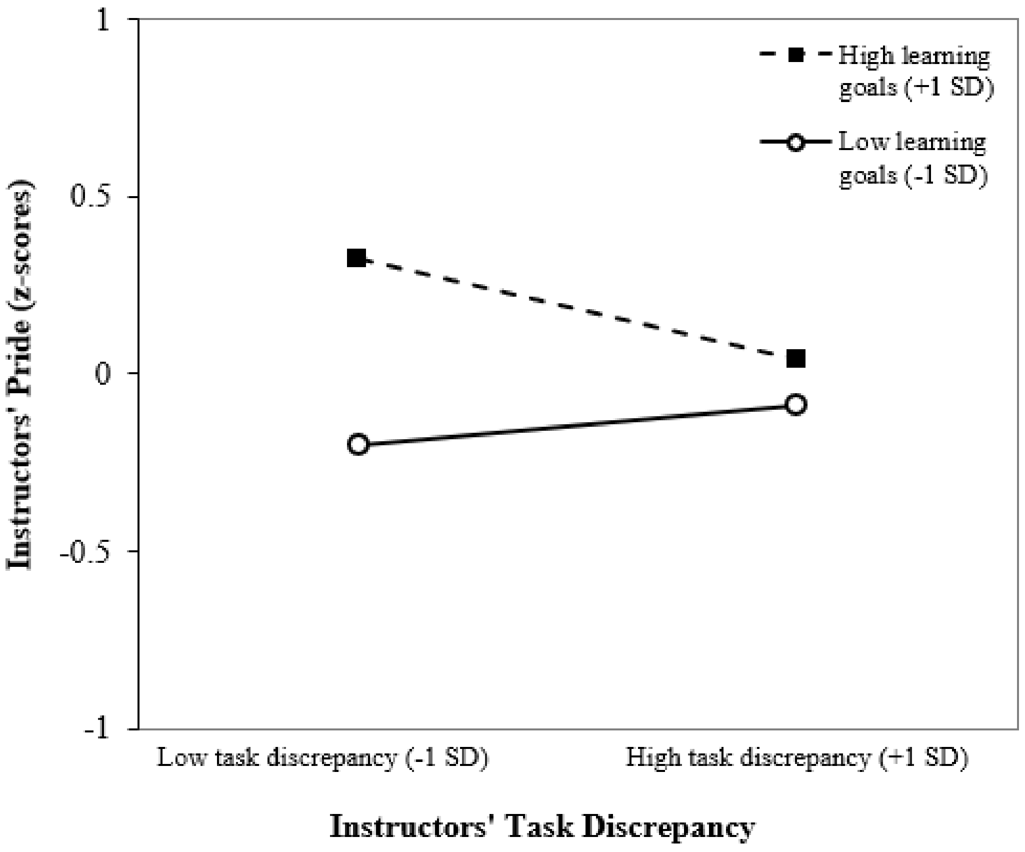

Supplementary Figure 1. Association between instructors' teaching-related task discrepancy and experience of pride, moderated by learning approach goals

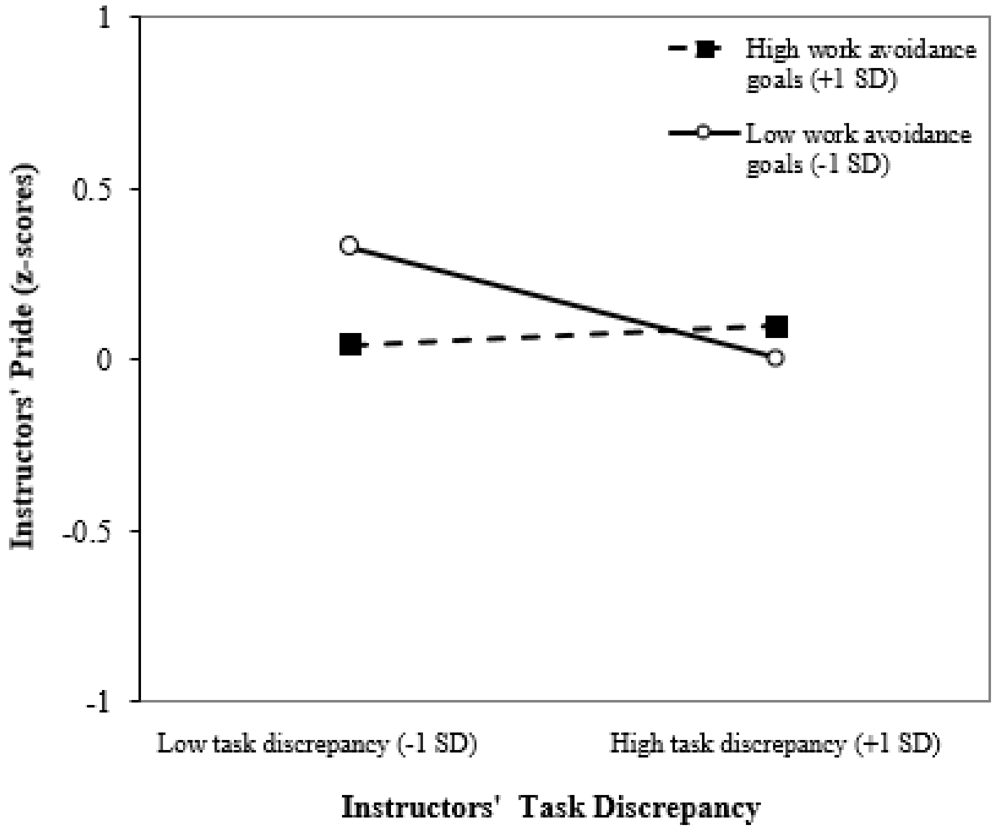

Supplementary Figure 2. Association between instructors' teaching-related task discrepancy and experience of pride, moderated by work avoidance goals.

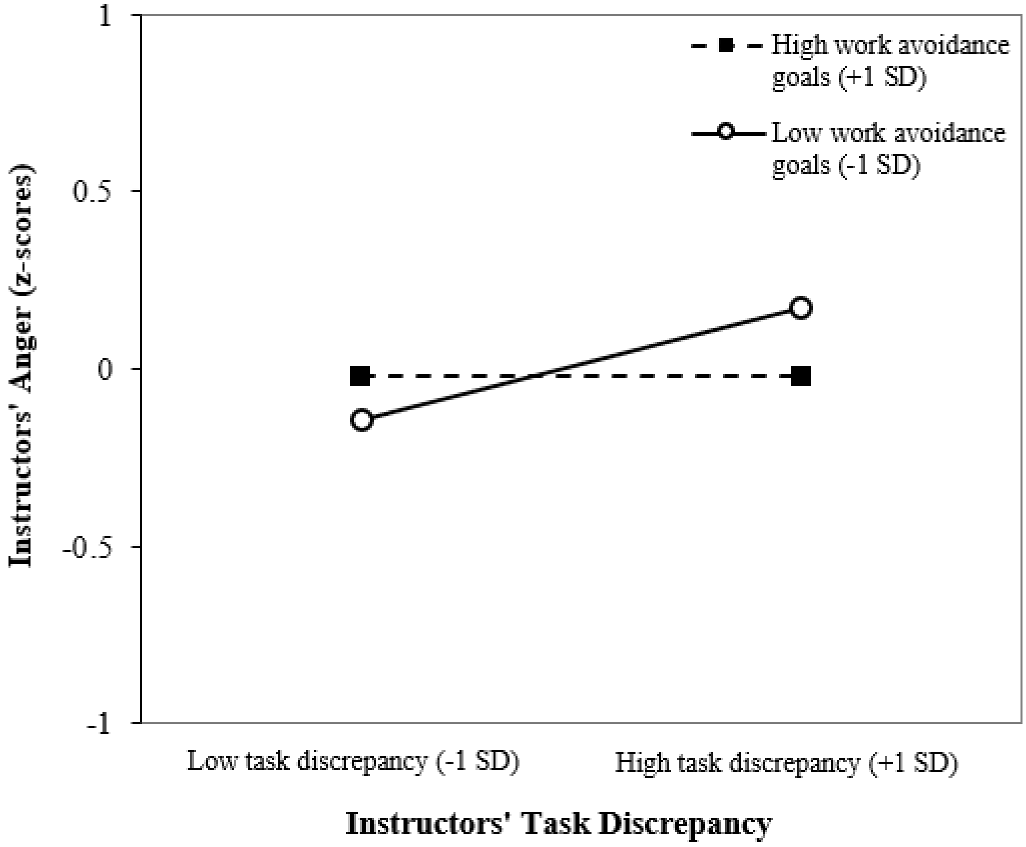

Supplementary Figure 3. Association between instructors' teaching-related task discrepancy and experience of anger, moderated by work avoidance goals.

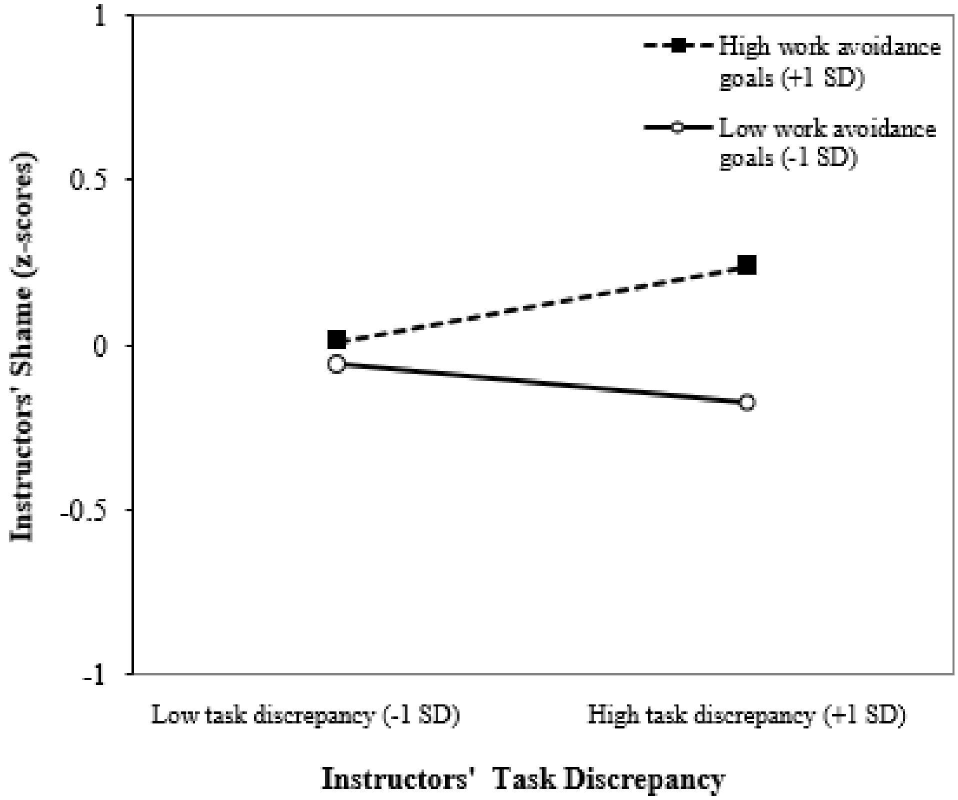

Supplementary Figure 4. Association between instructors' teaching-related task discrepancy and experience of shame, moderated by work avoidance goals.
